# Impact of Multimorbidity on Symptoms of Depression, Anxiety, and Stress in Older Adults: Is There a Sex Difference?

**DOI:** 10.3389/fpsyg.2021.762310

**Published:** 2021-12-21

**Authors:** Huang Lin, Shujuan Xiao, Lei Shi, Xiao Zheng, Yaqing Xue, Qilong Yun, Ping Ouyang, Dong Wang, Hong Zhu, Chichen Zhang

**Affiliations:** ^1^Shool of Public Health, Southern Medical University, Guangzhou, China; ^2^School of Health Management, Southern Medical University, Guangzhou, China; ^3^The First School of Clinical Medicine, Southern Medical University, Guangzhou, China; ^4^Tandon School of Engineering, New York University, New York, NY, United States; ^5^Department of Health Management, Nanfang Hospital, Southern Medical University, Guangzhou, China; ^6^Institute of Health Management, Southern Medical University, Guangzhou, China

**Keywords:** multimorbidity, depression, anxiety, stress, sex difference

## Abstract

**Introduction:** Multimorbidity has become a key issue in the health care sector globally, and it can also lead to psychological distress in older adults. This study aimed to assess the impact of multimorbidity on depression, anxiety, and stress symptoms and identify whether there is a sex difference in these associations.

**Methods:** A cross-sectional study using a multistage random sampling method was conducted among 3,266 older adults in China. Multiple linear regression models were used to estimate the independent associations between multimorbidity and depression, anxiety, and stress symptoms. Furthermore, interaction analysis was employed to investigate the interaction effect of multimorbidity and sex on depression, anxiety, and stress symptoms.

**Results:** A total of 3,250 participants aged 60 years and older were included in this study. Our findings suggest that multimorbidity is strongly positively associated with depression, anxiety, and stress symptoms. In addition, the positive relations between multimorbidity and depression, anxiety, and stress symptoms are stronger for older female than male adults.

**Conclusion:** Old adults with multimorbidity are more likely have depression, anxiety, and stress symptoms. This study offers new insight for the mental health from the perspective of multimorbidity among older people, implies that encouraging the accessibility of treatment for multimorbidity in older people with different sex may be effective in promoting mental health in China.

## Introduction

Aging is one of the most prominent features of recent global population dynamics. China has the largest population of older adults in the world. According to the National Bureau of Statistics of the People’s Republic of China, 253 million people aged 60 years and older accounted for 18.1% of the total population at the end of 2019 (Wang S. et al., 2020). The accelerated aging of the population has brought several problems and challenges to both economic and social development. For older individuals, aging presents not only losses in physiological function but also an increased threat of mental health problems (World Health Organization [WHO], 2017). With changes in lifestyle and social roles, older adults are at a high risk of various mental health problems (Yang et al., 2020). More than 20% of older adults suffer from mental or neurological disorders (excluding headache disorders) (Fang et al., 2019). A report by the China Aging Development Foundation in 2018 showed that 63% of older Chinese adults often feel lonely, with 62% often experiencing stress or depression (China Aging Development Foundation, 2018). In addition, previous studies have shown that mental disorders play a prime role in suicide attempts in rural China, and almost all older adult suicide victims have preceding mental disorders (Liu et al., 2018). Russ et al. (2012) found a dose-response association between psychological distress across the full range of severity and an increased risk of mortality, which illustrates the potential harm from psychological distress. However, attention should be paid to whether the mental health of patients with multimorbidity, is worse than that of older adults without multimorbidity.

Multimorbidity has become a key issue in the healthcare sector globally. The concept of multimorbidity is internationally accepted as being the simultaneous presence of two or more chronic diseases (Dattalo et al., 2017). Studies in Western countries have shown that 81% of Americans aged 65 years and older and 50% aged 45–65 years have multimorbidity. A cross-sectional survey conducted in southern China showed that 47.5% of older adults suffered from multimorbidity (Wang et al., 2014). The predominantly incurable nature of multimorbidity has adverse effects throughout life (Demirer et al., 2021). Older individuals with multimorbidity require additional support and medical services, incur higher healthcare costs, and are often at a higher risk of hospitalization (Goldberg et al., 2020). Recently, an increasing number of studies have focused on the effect of multimorbidity on psychological distress. One previous study has shown that the prevalence of mental illness increases with the number of chronic conditions, suggesting that living with multimorbidity might affect patients’ psychological well-being, not merely their physical well-being (Gunn et al., 2012). Another previous study found a significant link between multimorbidity and depressive symptoms, with participants who had multimorbidity having a higher risk of depressive symptoms than those without a chronic condition (Yao et al., 2020). Another study showed that three or more chronic diseases caused a 2.30-fold increase in elevated anxiety (Gould et al., 2016). Substantial evidence has also shown that patients with multimorbidity have a higher prevalence of stress and depression in other countries (Pruchno et al., 2016; Sakakibara et al., 2019). However, in China, little is known about the impact of multimorbidity on mental health compared to a single chronic disease (Jiang et al., 2020). Previous studies concerning the association between different chronic conditions and mental health have mostly focused on a single condition or on a number of conditions. Based on the above, this study aimed to assess the association between multimorbidity and developing symptoms of depression, anxiety, and stress in older adults in China.

Furthermore, sex is an important factor affecting health status (Wells et al., 2017). Previous evidence has suggested that sex differences exist in psychological distress and multimorbidity (Violan et al., 2014; Agur et al., 2016; Maestre-Miquel et al., 2021). Females in China have lower salaries and education levels, less social support, fewer social activities, and greater rates of widowhood than males, all of which can contribute to psychological symptoms (Ma et al., 2008). One explanation is that older female patients are regarded as a disadvantaged group in society, who often suffer from negative emotions and loneliness. Furthermore, the higher risk of depression and anxiety among women has been primarily linked to sex differences in biological susceptibility, genetic and hormonal factors, as well as sex differences in physiological stress responsivity or exposure to environmental risk factors, including higher exposure to sociocultural stressors, over the last decades (Hyde and Mezulis, 2020; Robb et al., 2020). Older male and female adults may differ not only in their levels of the multimorbidity and psychological distress but also in the effect of multimorbidity on psychological distress. However, the results of sex differences in the relationship between multimorbidity and mental health have been inconsistent. Recent research has identified that older adults in Taiwan showed no significant sex differences between chronic illness and mental health (Luo et al., 2018). However, a study of older adults in Shandong Province indicated that women with multimorbidity had a higher risk of mental health problems than men with multimorbidity (Jiao et al., 2020). Older females who effectively take care of their families have little time to participate in social activities, which places a premium on their spare time. Therefore, in this study, we aimed to explore whether there is a sex difference in the association between multimorbidity and depression, anxiety, and stress symptoms under the Chinese context. A better understanding of differences between the sexes regarding this factor in the context of China will help identify possible interventions addressing disparate risks of the symptoms of depression, anxiety, and stress.

Given the adverse effects of depression, anxiety, and stress symptoms, the aim of this study was to preliminarily explore the relationship between multimorbidity and depression, anxiety, and stress, and to determine whether there is a sex difference in these associations in older adults aged 60 years and above. From a theoretical perspective, we proposed the following hypotheses: Hypothesis 1: Multimorbidity has a negative effect on the mental health of older adults. Hypothesis 2: There are sex differences in the effects of multimorbidity on the depression, anxiety, and stress symptoms of older adults. Understanding the sex difference in the relationship between multimorbidity and depression, anxiety, and stress symptoms is crucial not only for theoretical advancement but also for promoting sex equality in active aging.

## Materials and Methods

### Sample and Participants

A questionnaire-based cross-sectional study was undertaken in Shanxi Province, in North China, which is composed of 11 cities. We used a multistage stratified cluster sampling method to select the participants. First, according to the order of districts (counties) on the government’s website, each district (county) was numbered in every city. Second, two districts (counties) were selected in each city using a random number table. Subsequently, two communities (administrative villages) were drawn from each district (county) in the same manner. Third, considering the different scales of each community (administrative village), one or two residential communities (natural villages) were again selected from each community (administrative village). Finally, we randomly selected older individuals who met the study criteria.

A total of 3,266 questionnaires were distributed, of which 3,250 respondents effectively completed the questionnaires; thus, the effective response rate was 99.51%. All study procedures were approved by the Ethics Committee of Shanxi Medical University (reference number: 2018LL177). All participants were informed of the purpose of this research and signed an informed consent form.

### Measures

#### Self-Made Questionnaire About Chronic Diseases

To categorize the participants as those with and those without multimorbidity, participants were asked to indicate whether they had ever been diagnosed with any of the following 26 chronic diseases (hypertension, diabetes, rheumatoid or rheumatoid arthritis, etc.) through a self-made chronic disease questionnaire. Information on chronic diseases was collected through self-reporting, which was based on the diagnostic evidence of medical records or prescriptions from doctors. Multimorbidity statuses were divided into two categories (non-multimorbidity and multimorbidity). Multimorbidity commonly defined as the presence of two or more chronic diseases (Nunes et al., 2016).

#### The 21-Item Depression, Anxiety, and Stress Scale

Depression, anxiety, and stress were measured using the Chinese version of the 21-item Depression, Anxiety, and Stress Scale (DASS-21), which may allow researchers to obtain more comprehensive research findings (Henry and Crawford, 2005). The DASS-21 has been extensively used with high validity and reliability in research on older adults (Abdul Manaf et al., 2016; Gholamzadeh et al., 2019). Each subscale includes seven items. Each item is scored on a four-point Likert scale (0 = does not apply to me at all and 3 = applies to me very much), with a higher score indicating more severe levels of distress. In the studied population, the Cronbach’s α of the total scale was 0.950; the Cronbach’s α values of the depression, anxiety, and stress subscales were 0.888, 0.845, and 0.876, respectively.

#### Potential Confounders

We identified potential confounders for multimorbidity and depression, anxiety, and stress symptoms based on previous studies (Min et al., 2016; Zhang et al., 2019; Li et al., 2020; Thapa et al., 2020). Potential confounders included sex, age, education level, marital status, empty nest status, sleep quality, and social participation. These factors were chosen because previous studies have shown that they are associated with multimorbidity and psychological distress.

### Statistical Analysis

Statistical analyses were performed using Stata 16.0 (Stata Corp., College Station, TX, United States), with results considered statistically significant when *p*-values were less than 0.05. The statistical description is expressed as frequencies (n) and proportion percentages (%). Differences between the sexes in the baseline characteristics were calculated using the Student’s *t*-test (for continuous variables) or chi-square test (for categorical variables). Analysis of variance was used to compare differences in multimorbidity, depression, anxiety, and stress scores. Subsequently, we used multiple linear regression models to see how the coefficients of multimorbidity change and estimate independent associations between multimorbidity and symptoms of depression, anxiety, and stress. To test the heterogeneity of sex between the two types of multimorbidity status and three types of psychological distress, we added the multiplicative interaction term (multimorbidity × sex) among older adults.

## Results

### Baseline Characteristics

There were 2265 (69.69%) participants without multimorbidity, and 985 (30.31%) participants with multimorbidity. The number of chronic diseases in multimorbidity participants ranged from two to nine, with the coexistence of two, three, and four chronic conditions accounting for 54.11, 26.19, and 11.17%, respectively. Table 1 shows the characteristics of the participants according to sex. Of the 3,250 participants, 46.62% were men.

**TABLE 1:** Participants’ characteristics.

Variables	All	Male *n* (%)	Female *n* (%)	*P*-value
Total	3250	1515 (46.62)	1735 (53.38)	
Depression score, mean (SD)	5.73 (7.40)	5.31 (7.25)	6.09 (7.51)	0.003^a^
Anxiety score, mean (SD)	6.46 (6.92)	6.00 (6.88)	6.86 (6.92)	<0.001^a^
Stress score, mean (SD)	6.96 (7.15)	6.38 (7.42)	7.46 (7.63)	0.002^a^
Multimorbidity status				0.009^b^
Non-multimorbidity	2265 (69.69)	1090 (71.95)	1175 (67.72)	
Multimorbidity	985 (30.31)	425 (28.05)	560 (32.28)	
Age				0.125^b^
60∼69	1769 (54.43)	796 (52.54)	973 (56.08)	
70∼79	1164 (35.82)	563 (37.16)	601 (34.64)	
≥80	317 (9.75)	156 (10.30)	161 (9.28)	
Educational level				<0.001^b^
No education	577 (17.75)	173 (11.42)	404 (23.29)	
Primary school	1062 (36.68)	428 (28.25)	634 (36.54)	
Junior high school	850 (26.15)	455 (30.03)	395 (22.77)	
High school/technical secondary school	474 (14.59)	282 (18.61)	192 (11.07)	
College/higher vocational	144 (4.43)	96 (6.34)	48 (2.77)	
Bachelor degree and above	143 (4.40)	81 (5.35)	62 (3.56)	
Marital status				<0.001^b^
Married	2482(76.36)	1236 (81.58)	1246(71.82)	
Unmarried	50 (1.54)	31 (2.05)	19 (1.10)	
Divorced	54 (1.66)	27 (1.78)	27 (1.56)	
Widowed	664 (20.44)	221 (14.59)	443 (25.52)	
Empty nest status				0.019^b^
Empty nest	1651 (50.80)	803 (53.00)	848 (48.88)	
Non-empty nest	1599 (49.20)	712 (47.00)	887 (51.12)	
Sleep quality				<0.001^b^
Good	2574 (79.20)	1253 (82.71)	1321 (76.14)	
Poor	676 (20.80)	262 (17.29)	414 (23.86)	
Social participation				<0.001^b^
Yes	2837 (87.29)	1275 (84.16)	1562 (90.03)	
No	413 (12.71)	240 (15.84)	173 (9.97)	

*SD, standard deviation.*

*^a^t-test.*

*^b^Chi-square test.*

### Comparison of Depression, Anxiety, and Stress Between Multimorbidity Status

Table 2 presents the results from the comparison of depression, anxiety, and stress scores for the multimorbidity status in female and male older adults separately. There were significant differences between the sexes in mean depression, anxiety, and stress scores for the multimorbidity status.

**TABLE 2:** Comparison of depression, anxiety, and stress between different multimorbidity status.

Variable	Non-multimorbidity	Multimorbidity	*P*-value
**Male**			
Depression score, mean ± SD	4.68 ± 6.96	6.92 ± 7.70	0.001^a^
Anxiety score, mean ± SD	5.30 ± 6.67	7.77 ± 7.11	0.015^a^
Stress score, mean ± SD	5.79 ± 7.28	7.90 ± 7.57	0.046^a^
**Female**			
Depression score, mean ± SD	5.32 ± 7.00	7.72 ± 8.26	<0.001^a^
Anxiety score, mean ± SD	5.85 ± 6.39	8.98 ± 7.50	<0.001^a^
Stress score, mean ± SD	6.64 ± 7.17	9.19 ± 8.26	<0.001^a^

*SD, standard deviation.*

*^a^t-test for differences of multimorbidity status.*

### Association Between Multimorbidity and Psychological Distress and Its Sex Differences

Table 3 summarizes the multiple linear regression models of psychological distress measured using the DASS-21 (supporting Hypothesis 1). In Model 1, we found that multimorbidity was positively associated with depression, anxiety, and stress scores and that the coefficient was significant. Model 2 included age, education, marital status, prevalence of empty nest syndrome, sleep quality, and social participation. After adding the variables, multimorbidity group remained positively associated with depression, anxiety, and stress scores. Nevertheless, we found that all the coefficients of the multimorbidity group declined compared with Model 1, but remained significant. Specifically, older adults with multimorbidity scored an average of 1.445, 2.036, and 1.664 more points than older adults without multimorbidity for depression, anxiety, and stress, respectively.

**TABLE 3:** The interaction effect between sex and multimorbidity on depression, anxiety, and stress symptoms among the older people (*n* = 3250).

Variables	Depression		Anxiety		Stress	
	Model 1	Model 2	Model 1	Model 2	Model 1	Model 2
**Multimorbidity status (Reference: Non-multimorbidity)**						
Multimorbidity	2.361***	1.445***	2.870***	2.036***	2.401***	1.664***
F	71.44	36.51	122.59	39.51	70.90	34.02
**Female (Reference: Male)**						
**Multimorbidity status × Sex**						
Multimorbidity × Female	3.035***	1.483***	3.670***	2.217***	3.395***	2.053***
F	26.24	31.93	44.72	34.61	28.55	29.91

*Model 1 was unadjusted; Model 2 was adjusted for age, education level, marital status, empty nest status, sleep quality, social participation.*

***P < 0.01.

**FIGURE 1:**
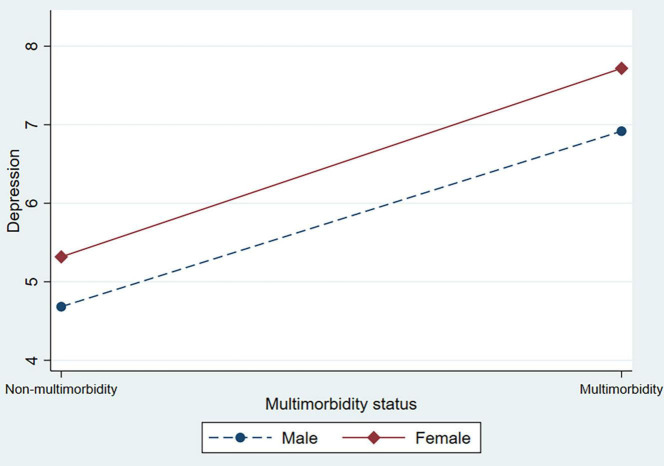
The interaction effect between multimorbidity status and sex on depression among older adults.

**FIGURE 2:**
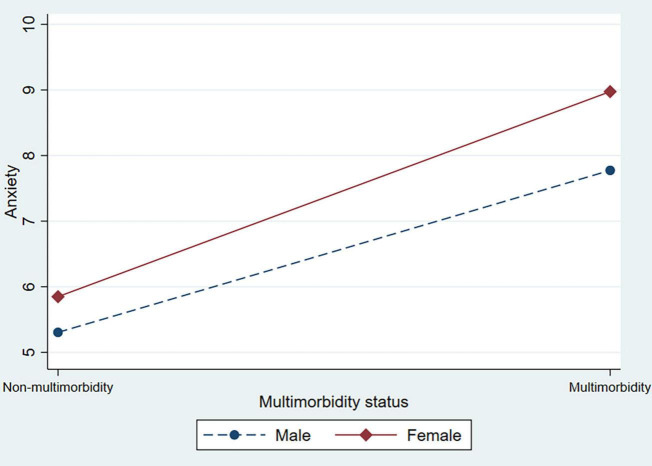
The interaction effect between multimorbidity status and sex on anxiety among older adults.

**FIGURE 3:**
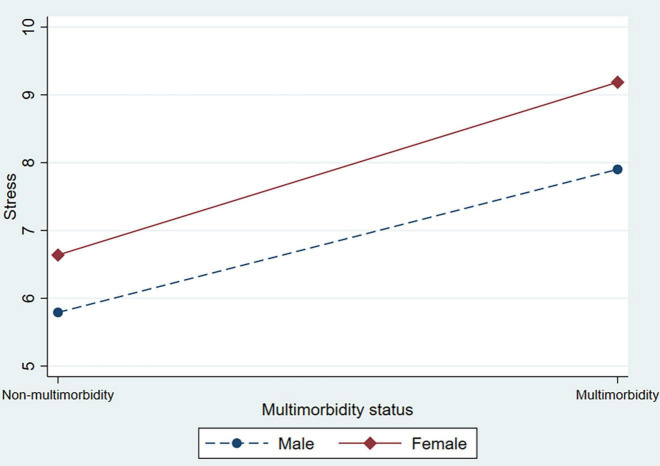
The interaction effect between multimorbidity status and sex on stress among older adults.

In Table 3, the interaction terms between multimorbidity status and sex were added to test the heterogeneity of sex (supporting Hypothesis 2). In Model 2, female respondents who had multimorbidity were positively associated with depression, anxiety, and stress scores. This indicated that the association between multimorbidity and psychological distress was particularly pronounced for female respondents. Figure 1 shows this interaction in the symptoms of depression. Figure 2 also shows the significant interaction between multimorbidity status and sex in anxiety symptoms. Figure 3 shows the significant interaction between multimorbidity status and sex in stress symptoms. Taken together, our findings suggest that symptoms of depression, anxiety, and stress among older male adults are more elastic in relation to multimorbidity than among older female adults.

## Discussion

China has undergone rapid population aging and there is a lack of empirical evidence examining the relationship between multimorbidity and depression, anxiety, and stress symptoms among older adults in China. In this study, we found that multimorbidity had a significant effect on symptoms of depression, anxiety, and stress after controlling for potential confounders, which is consistent with previous research (Gunn et al., 2012; Demirer et al., 2021). In addition, the positive relations between multimorbidity and depression, anxiety, and stress symptoms are stronger for older female than male adults. This implies that public health interventions to improve mental health should be sensitive to sex differences, and focus more on older female adults.

Results from a longitudinal study have confirmed that multimorbidity increases the risk of mental health disorders among older individuals compared to those without a chronic disease (Yan et al., 2021). Multiple linear regression analysis also showed that multimorbidity was positively associated with depression, anxiety, and stress symptoms in the current study. Our findings were also consistent with previous research, which found that multimorbidity and depression co-vary in a dose-dependent manner, with an increasing number of chronic diseases associated with an increased likelihood of depression and other mental health conditions (Barnett et al., 2012). Our study made a further contribution by comparing the presence of depression, anxiety, and stress symptoms across multimorbidity status. One reason for this situation may be the possibility of increased physical discomfort and increased psychological burden caused by multimorbidity; another reason might be that the disease involves greater financial costs (Chen et al., 2019). The more chronic conditions older adults have simultaneously, the greater the burden of disease, which leads to a high incidence of psychological distress. When treating multimorbidity, clinicians should pay attention to mental health and provide timely psychological intervention for older adults. Therefore, it is necessary to ensure the accessibility of treatment for multimorbidity and psychological distress among older adults, which can effectively improve their physical and mental health.

We found that older females suffered from the positive effects of multimorbidity on depression, anxiety, and stress symptoms, even after adjusting for other potential confounders. Taking sex into account is essential for understanding health problems. In line with our results, another study showed that sex had a significant effect on the association between multimorbidity and mental health, and women with multimorbidity had a higher risk of mental health problems (Jiao et al., 2020). Previous studies showed that women were at a high risk of psychological distress (Donner and Lowry, 2013; Lin et al., 2021). There are several reasons for the great impact of multimorbidity on depression, anxiety, and stress symptoms in female older adults in China. One possible explanation for sex differences in multimorbidity is that women who live in illness-causing poverty may be more susceptible to disease than males. Furthermore, economic misery may increase the likelihood of women experiencing psychological distress as a result of multimorbidity (Jiao et al., 2020). Another possible explanation is that females’ physical and psychological structures are more vulnerable to external factors (Zhang et al., 2019). Traditional Chinese culture is one major aspect that may explain the difference. In China, traditional culture holds that women should handle most of the family’s domestic issues, while males are solely responsible for participation in community-related organizations (Wang Y. et al., 2020). The sex role split of the family is more apparent in older individuals, due to the effect of traditional Chinese culture. Older females who effectively take care of their families have little time to participate in social activities, which places a premium on their spare time. Therefore, it is difficult for them to relieve their life stress, making them prone to psychological distress. As a result, women with multimorbidity are more likely to suffer from mental health problems than men. Sex analysis is an important way to understand the physical condition of multimorbidity in women and implement targeted interventions to prevent and treat physical and mental illnesses.

Overall, our findings have important implications for public health policy and planning. It is important to introduce targeted interventions to prevent multimorbidity and improve mental health among older adults. In response to the effect of multimorbidity on psychological distress, it is important to establish a safety net for the early identification and intervention in older individuals with multimorbidity. When developing psychological interventions for older adults, sex differences should be considered, with a focus on the older female population. Families and communities, especially those groups that often interact with older adults—family members, relatives, friends, and neighbors—need a collaborative approach because they can identify high-risk older people at an early stage for prevention. Importantly, clinicians should help patients with multimorbidity build confidence in their treatment and relieve negative emotions over time.

However, this study had several limitations. First, because the data used in this study were based on self-reports, recall bias could exist. Second, due to the cross-sectional design, the association between multimorbidity and depression, anxiety, and stress cannot be interpreted as cause and effect. To clarify the relationship between multimorbidity status and symptoms of depression, anxiety, and stress, longitudinal studies will be necessary in further research. Third, our sample consisted of older adults living in Shanxi Province in North China; further study is required to find out whether the results are applicable to older people across the whole of China.

## Conclusion

We found that older adults with multimorbidity are at a high risk of psychological distress, with an additional suggestion of a higher susceptibility to psychological distress in female older adults. Specifically, the interaction results suggest a disadvantage from psychological distress for women. Our results suggest that sex should be considered along with multimorbidity when selecting target populations for mental health interventions. It is important to create a positive psychology promotion system at the sex level among older adults that is conducive to promoting sex equality.

## Data Availability Statement

The raw data supporting the conclusions of this article will be made available by the authors, without undue reservation.

## Ethics Statement

All study procedures were approved by the Ethics Committee of Shanxi Medical University (reference number: 2018LL177). All participants were informed of the purpose of this research and signed an informed consent form. The patients/participants provided their written informed consent to participate in this study.

## Author Contributions

HL and SX conceived the idea and drafted the manuscript. SX and QY participated in data collection and statistical analysis. DW, HZ, and PO edited the manuscript. CZ, LS, XZ, and YX gave many valuable comments on the draft and polished it. All authors have read and approved the manuscript.

## Conflict of Interest

The authors declare that the research was conducted in the absence of any commercial or financial relationships that could be construed as a potential conflict of interest.

## Publisher’s Note

All claims expressed in this article are solely those of the authors and do not necessarily represent those of their affiliated organizations, or those of the publisher, the editors and the reviewers. Any product that may be evaluated in this article, or claim that may be made by its manufacturer, is not guaranteed or endorsed by the publisher.
